# DEEMD-SPP: A Novel Framework for Emotion Recognition Based on EEG Signals

**DOI:** 10.3389/fpsyt.2022.885120

**Published:** 2022-04-27

**Authors:** Jing Chen, Haifeng Li, Lin Ma, Frank Soong

**Affiliations:** ^1^School of Computer Science and Technology, Faculty of Computing, Harbin Institute of Technology, Harbin, China; ^2^Speech Group, Microsoft Research Asia, Beijing, China

**Keywords:** EEG, emotion recognition, affective computing, DEEMD, SPP-net

## Abstract

Electroencephalography (EEG) is one of the most widely-used biosignal capturing technology for investigating brain activities, cognitive diseases, and affective disorders. To understand the underlying principles of brain activities and affective disorders using EEG data, one of the fundamental tasks is to accurately identify emotions from EEG signals, which has attracted huge attention in the field of affective computing. To improve the accuracy and effectiveness of emotion recognition based on EEG data, previous studies have successfully developed numerous feature extraction methods and classifiers. Among them, ensemble empirical mode decomposition (EEMD) is an efficient signal decomposition technique for extracting EEG features. It can alleviate the mode-mixing problem by adding white noise to the source signal. However, there remain some issues when applying this method to recognition tasks. As the added noise cannot be filtered completely, spurious modes are generated due to the residual noise. Therefore, it is crucial to perform intrinsic mode function (IMF) selection to find the most valuable IMF components that represent brain activities. Furthermore, the number of decomposed IMFs is various to different original signals, thus how to unify feature dimensions needs better solutions. To solve these issues, we propose a novel forecasting framework, named DEEMD-SPP, to identify emotions from EEG signals, based on the combination of denoising ensemble empirical mode decomposition (DEEMD) and Spatial Pyramid Pooling Network (SPP-Net). First, DEEMD is proposed to decompose the EEG signals, which effectively eliminates residual noise in the IMFs and selects the most valuable IMFs. Second, time-domain and frequency-domain features are extracted from the selected IMFs. Finally, SPP-net is employed as the classifier to recognize emotions, which can effectively transform various-sized feature maps into fixed-sized feature vectors through the pyramid pooling layer. The experimental results demonstrate that our proposed DEEMD-SPP framework can effectively reduce the effect of spike-in white noise, accurately extract EEG features, and significantly improve the performance of emotion recognition.

## Introduction

Emotions are human responses to environmental objects or events (1), and emotion status is a widely measured phenotypical trait in psychological and psychiatric researches (2). For example, precise estimation of emotion status has become a fundamental task in many studies of cognitive and affective disorders (3). In the last decades, there has been a growing appreciation for the important contribution of physiological signal measurement technologies in emotion detection in the field of affective computing (4). Among the various types of physiological measurements, the EEG technology can directly capture the electrical activity of the human brain, and it can provide a cheap, portable, and easy-to-use solution for identifying emotions (5). The development of EEG has powered the research area of emotion recognition and increased the potential of investigating the neural underpinnings of emotion. Although EEG has provided an unparalleled opportunity to investigate human emotions and brain activities, how to accurately extract the valuable features hidden in the EEG signals remains challenging.

With the development of experimental instruments, the EEG data has been accumulated and aided the psychological and biological research together with multimodal omics (6). A series of computational methods and tools have been developed to deal with such data challenges (7–9). Particularly, a variety of signal analysis methods have been proposed to capture the characteristics of the EEG signals (10, 11). Among them, time-frequency analysis methods are found efficient in discovering the complex hidden features underlying EEG signals (12). These methods analyze the characteristics of the signal in both the time domain and frequency domain, simultaneously. The widely applied time-frequency analysis techniques rely on short-time Fourier transform (STFT), wavelet transform (WT), and their variations (13, 14). The limitation of STFT is the conflictive resolution of time and frequency. The frequency resolution will be sacrificed if time resolution is improved, and vice versa. Wavelet-based methods have advantages in time-frequency localization. However, the selection of wavelet kernel function is usually not objective, which largely affects the quality of EEG signal decomposition.

More recently, a new data-driven time-frequency analysis technique, called empirical mode decomposition (EMD), has been proposed for the analysis of non-linear and non-stationary signals (15). EMD is a robust decomposition algorithm. It is capable of decomposing complex and non-linear multi-component signals into a finite number of intrinsic mode functions (IMFs). IMFs are considered as a set of oscillation components of original EEG signals. Traditionally, EEG frequency bands are described as a fixed range of wave frequencies and amplitudes over a time scale. The commonly used bands are gamma (30–100 Hz), beta (14–29 Hz), alpha (8–13 Hz), theta (4–7 Hz), and delta (1–3 Hz). Different from the traditional frequency bands, the mode of each IMF corresponds to a specific frequency band containing the natural oscillatory contents of the original signal. Many researchers have investigated the properties of IMFs from EEG signals (16, 17). And they found different IMF scales bearing significant local information were associated with the EEG activities (18). Features extracted from IMFs have been used in the detection of diseases, such as schizophrenia (19, 20) and epileptic seizures (21, 22).

EMD has been successfully applied to observe and analyze EEG signals. However, it suffers from a “mode-mixing” problem (23). Mode mixing refers to the situation when different oscillating components may present in one IMF or similar oscillations may appear in different IMFs. The ensemble empirical mode decomposition (EEMD) has been proposed to overcome this problem (24). This method adds random white noise into the original EEG signal in several trials. The final IMF of EEMD is obtained by averaging the IMF related to *N* trials. EEMD can alleviate the mode-mixing problem, but it also induces biases. If the number of the ensemble is too small, or the noise amplitude is too large, the IMF components are biased by the added noises. Therefore, it is crucial to perform IMF selection to find the most valuable IMF components that can represent brain activities. Islam et al. (25) presented a model to select optimal IMF of EMD for diagnosing the sleep disorder based on EEG signal. They extracted Shannon entropy, spectral entropy, standard deviation, skewness, and kurtosis of each IMF as improved features for the task of disease classification. They evaluated the performance of different IMFs and found the optimal IMFs. The experiments revealed that the selected IMFs performed better for sleep disorder diagnosis.

Furthermore, there remains a common issue in most EMD-based approaches when applying these methods to recognition tasks: the number of decomposed IMFs is various to different original signals. How to unify feature dimensions needs better solutions. Several previous studies chose a fixed number of IMFs. Zhuang et al. (26) extracted features from the first five IMFs for emotion recognition from EEG signals. Shahnaz and Hasan (27) sorted the IMFs in descending order by temporal energy content and choose the top three of them as the dominant IMFs. Riaz et al. (17) proposed a method for the detection of seizures and epilepsy based on the EEG signals. They selected the first three IMFs, then extracted the temporal and spectral characteristics of these IMFs. Although these methods were able to achieve their goals, the arbitrary selection of IMFs could result in information loss and affect downstream analyzes. Particularly, previous studies have shown that not all of the IMF components are equally important in the EEG analysis, and the top IMFs are sometimes even noise-dominant components (25).

In this article, we propose a novel framework, named DEEMD-SPP, to address these challenges and use it to improve the performance of EEG-based emotion recognition. Our contribution is 2-fold: (1) DEEMD is proposed to decompose the EEG signals. It effectively eliminates residual noise in the IMFs and selects the most valuable IMFs. (2) SPP-net can process arbitrarily sized input and aggregate information at a multi-level. Experiments on a dataset of speech-evoked EEG responses demonstrated our proposed framework can effectively improve the accuracy of EEG-based emotion recognition.

## Materials and Methods

**Figure 1:**
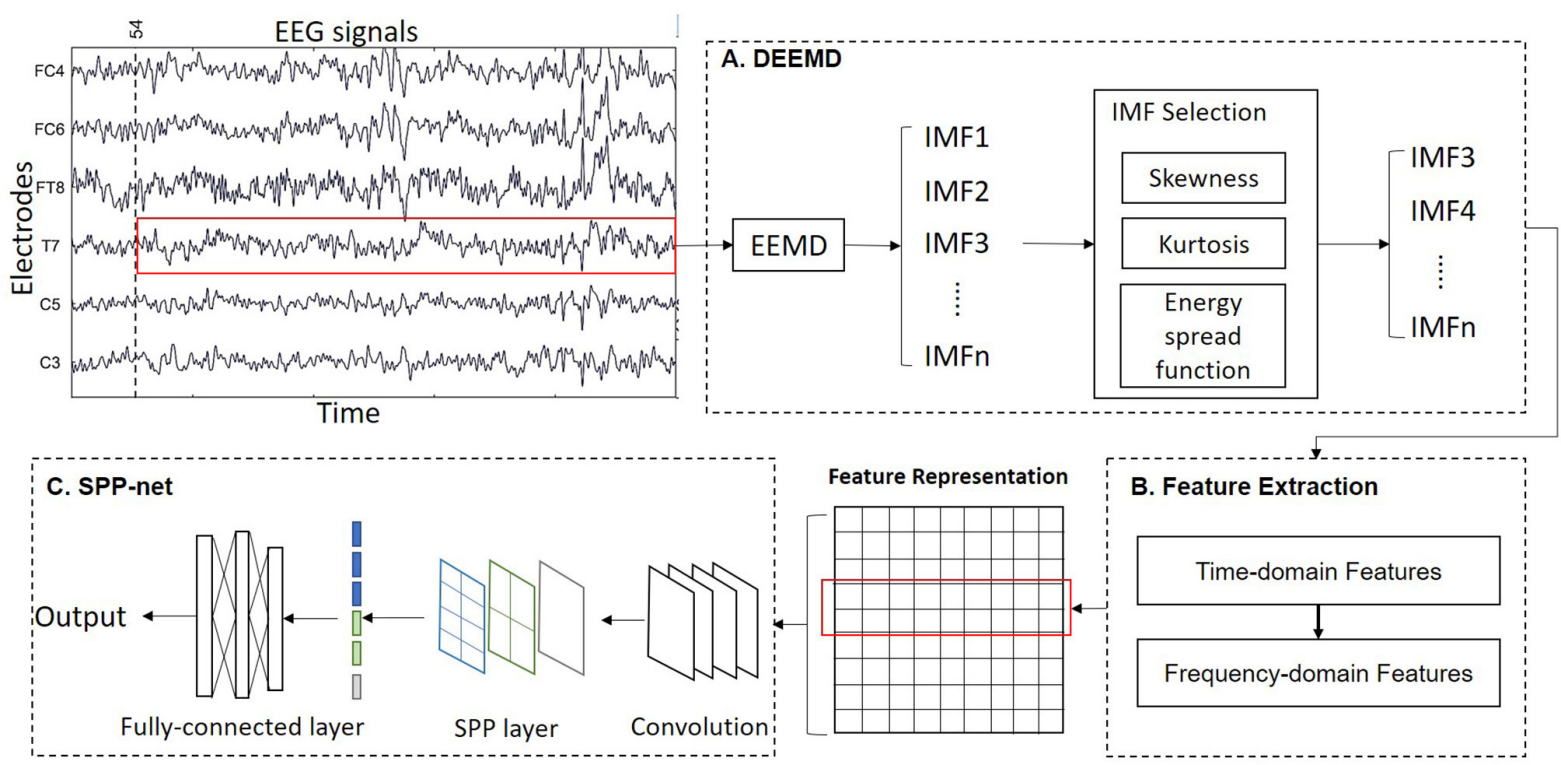
Overall scheme of the DEEMD-SPP. **(A)** DEEMD algorithm for signal decomposition. **(B)** Feature extraction of IMFs. **(C)** SPP-net for emotion recognition.

We propose a novel algorithm called DEEMD-SPP to predict emotion based on EEG signals. The framework of DEEMD-SPP is shown in Figure 1 and it contains three steps. First, we propose DEEMD to decompose the EEG signal of each electrode (Figure 1A). Specifically, we apply EEMD to decompose EEG signals into a series of IMFs. We propose an evaluation criterion with three indicators to select IMFs that contain significant information. These indicators are derived from the white noise through EMD and are tested against the results produced on numerically generated white noises. Second, time domain and frequency domain features are extracted from the selected IMFs (Figure 1B). These features give a rich clue about the physiology of the EEG signal. The features of all electrodes are concentrated to a feature representation. Third, we apply a spatial pyramid pooling net (SPP-net) to further extract higher-level features from the feature representation obtained in the last step and perform emotion recognition (Figure 1C). SPP-net can process arbitrarily sized input and aggregate information at a multi-level. By using the proposed framework, we can overcome the limitations of EEMD in the case of recognition tasks.

### Denoising Ensemble Empirical Mode Decomposition

#### EEMD Algorithm

Intrinsic mode function (IMF) is introduced by Huang et al. (28) for analyzing non-linear and non-stationary time series signals. An IMF has to satisfy two conditions: (1) The number of extrema equals the number of zero-crossing or differs at most by one. (2) At any point, the mean value of the envelope defined by the local maxima and local minima is zero. With this definition, each IMF represents one mode of oscillation with the same timescale. And they are both amplitude and frequency modulated.

The empirical mode decomposition (EMD) method was proposed to derive IMFs. However, the mode mixing problem is one of the limitations of EMD. The ensemble empirical mode decomposition (EEMD) is developed to overcome this problem. EEMD defines the true IMF components as the mean of an ensemble of EMD trials. Each trial consists of the signal and generated instances of white noise. More particularly, the algorithm is described below:

Given a discrete signal *x* (*t*) (t = 1, 2,…, n), it can be decomposed in the following steps through a sifting process:

Step 1: Add white noise series *w*(*t*) to the original signal *x* (*t*),
  (1) $$\begin{array}{l} y\left(t\right)=x\left(t\right)+w\left(t\right) \end{array}$$
Step 2: Identify all local maxima and minima of the signal *y* (*t*);
Step 3: Connect all maxima and minima points to produce the upper (*e*_max_(*t*)) and lower (*e*_min_(*t*)) envelops by a cubic spline line, respectively.
Step 4: Calculate the mean value *m* (*t*) between two envelops and define the difference between *y* (*t*) and *m*(*t*) as *h*(*t*):
  (2) $$\begin{array}{l} m\left(t\right)=\left(e_{\max}\left(t\right)+e_{\min}\left(t\right)\right)/2 \end{array}$$
  (3) $$\begin{array}{l} h\left(t\right)=y\left(t\right)-m\left(t\right) \end{array}$$
  If *h*(*t*) meets the two conditions of IMF, *h*(*t*) is denoted as the first IMF component *c*_1_(*t*). If *h*(*t*) is not an IMF, replace *y* (*t*) with *h*(*t*), and iterate steps 2-4 until *h*(*t*) meets the conditions.
Step 5: Take the residue *r*(*t*) = *y* (*t*) − *c*_1_(*t*) as new data and subject to the same sifting process steps 2-4 for the next IMF. The sifting process is stopped when *r*(*t*) becomes a monotone function. The signal *y* (*t*) is decomposed into IMF components *c*_*i*_ (*t*), *i* = 1, 2, 3…., *m* and a residual signal *r*_*m*_(*t*). *m* is the number of IMFs.
Step 6: Repeat the above 5-steps *N* times by adding different white noise series each time and obtain the corresponding IMF components. Average the above results to get the final IMF component:
  (4) $$\begin{array}{l} imf_{i}\left(t\right)=\frac{1}{N}\sum_{j=1}^{N}c_{ij}\left(t\right) \end{array}$$

The original signal *x* (*t*) can be reconstructed using the extracted intrinsic modes and the residue signal:

(5) $$\begin{array}{l} x\left(t\right)=\sum_{i}^{m}imf_{i}\left(t\right)+r_{m}\left(t\right) \end{array}$$

The previous studies (29–31) demonstrate that EEMD is capable of better separation of the extracted signal modes and drastically reduces the influence of the mode-mixing problem.

#### DEEMD Algorithm

EEMD can enhance the stability of the EMD algorithm by adding appropriate noise. However, the resulting IMFs derived from EEMD would inevitably be contaminated by the added noise especially when the number of the ensemble was relatively low. The residual noises generate spurious modes. In this article, we proposed DEEMD for reducing the effect of spike-in white noise by automatically selecting the valuable IMFs. We first introduce the criteria for IMF selection and then detail the procedure of DEEMD.

(1) Criterion for IMF selection

**Figure 2:**
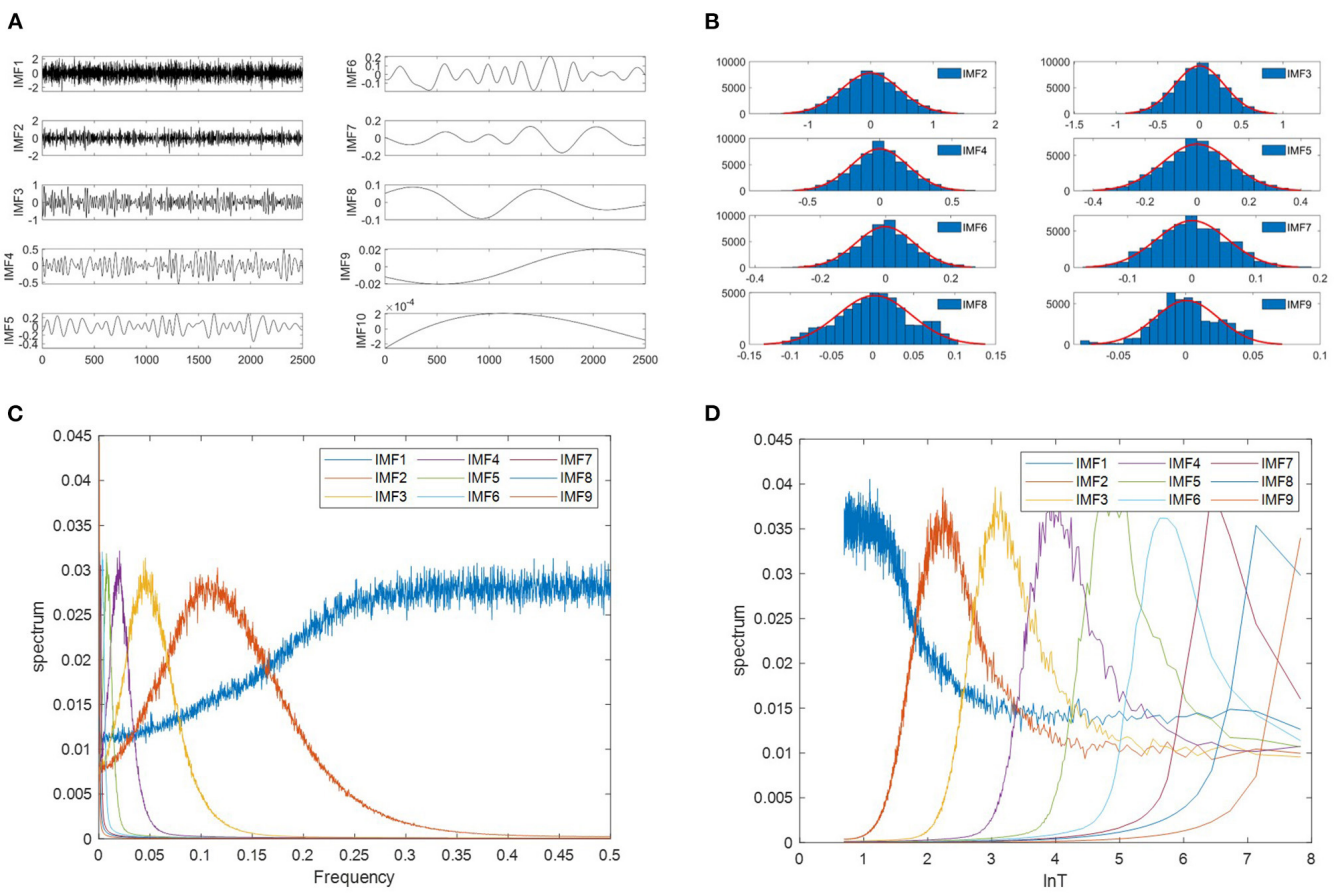
Properties of IMFs derived from white noise using EMD. **(A)** The waveforms of the first 10 IMFs decomposed from a white noise signal. The length of the white noise is 2,500 points. **(B)** The probability distribution function(PDF) of IMF follows a normal distribution. **(C)** EMD equivalent filters. 1,000 independent white noises of 2,500 points each have been generated, and average spectra of the nine IMFs are plotted as a function of normalized frequency. **(D)** The Fourier spectra of IMFs as a function of the logarithm of the period. The samples are the same with **(C)**.

Wu and Huang (32) explored the characteristics of white noise using the EMD method. They studied the relationship between the energy density and the mean period of the IMFs. Analytic expressions of the relationship were derived and tested against the results produced by Monte Carlo method on a numerically generated random noise. Figure 2A presents an example of the top ten IMF components decomposed from white noise. They found the following empirical facts: (a) IMF components have mean periods approximately twice the value of the previous component. (b) IMF components are normally distributed (Figure 2B). (c) The IMF1 corresponds essentially to a half-band highpass filter, other IMFs can be interpreted as a filter bank of the overlapping bandpass filters (Figure 2C). (d) The Fourier spectra of IMFs are identical in shape and cover the same area on the semi-logarithmic period scale (Figure 2D).

These characteristics can provide the criteria for determining which IMFs contain statistically significant information and which IMFs are purely noise (32, 33). Considering the amplitude and frequency properties of the IMFs derived from white noise, an evaluation criterion with three indicators is proposed in this section. These three indicators are skewness, kurtosis, and the energy-density spread function. The following are the details of the criterion.

As IMF components of white noise are normally distributed, two numerical measures– skewness and kurtosis – can be used to test the shape of IMF.

- Skewness is a measure of the symmetry of the data around the mean. It is the standardized third central moment of the probability distribution. If skewness is negative, the data spread out more to the left of the mean than to the right. If skewness is positive, the data spread out more to the right. The skewness of the normal distribution (or any perfectly symmetric distribution) is zero. It is given as follows:
  (6) $$\begin{array}{l} \text{S}=\frac{E{\left[X-E\left(X\right)\right]}^{3}}{{\left\{E{\left[X-E\left(X\right)\right]}^{2}\right\}}^{3/2}} \end{array}$$
- Kurtosis is a measure of whether the data are peaked or flat relative to a normal distribution and it is the standardized fourth central moment of the probability distribution. The kurtosis of the normal distribution is 3. Distributions that are more outlier-prone than the normal distribution have kurtosis >3; distributions that are less outlier-prone have kurtosis <3. It is given as follows:
  (7) $$\begin{array}{l} \text{K}=\frac{E{\left[X-E\left(X\right)\right]}^{4}}{{\left\{E{\left[X-E\left(X\right)\right]}^{2}\right\}}^{2}} \end{array}$$

**Figure 3:**
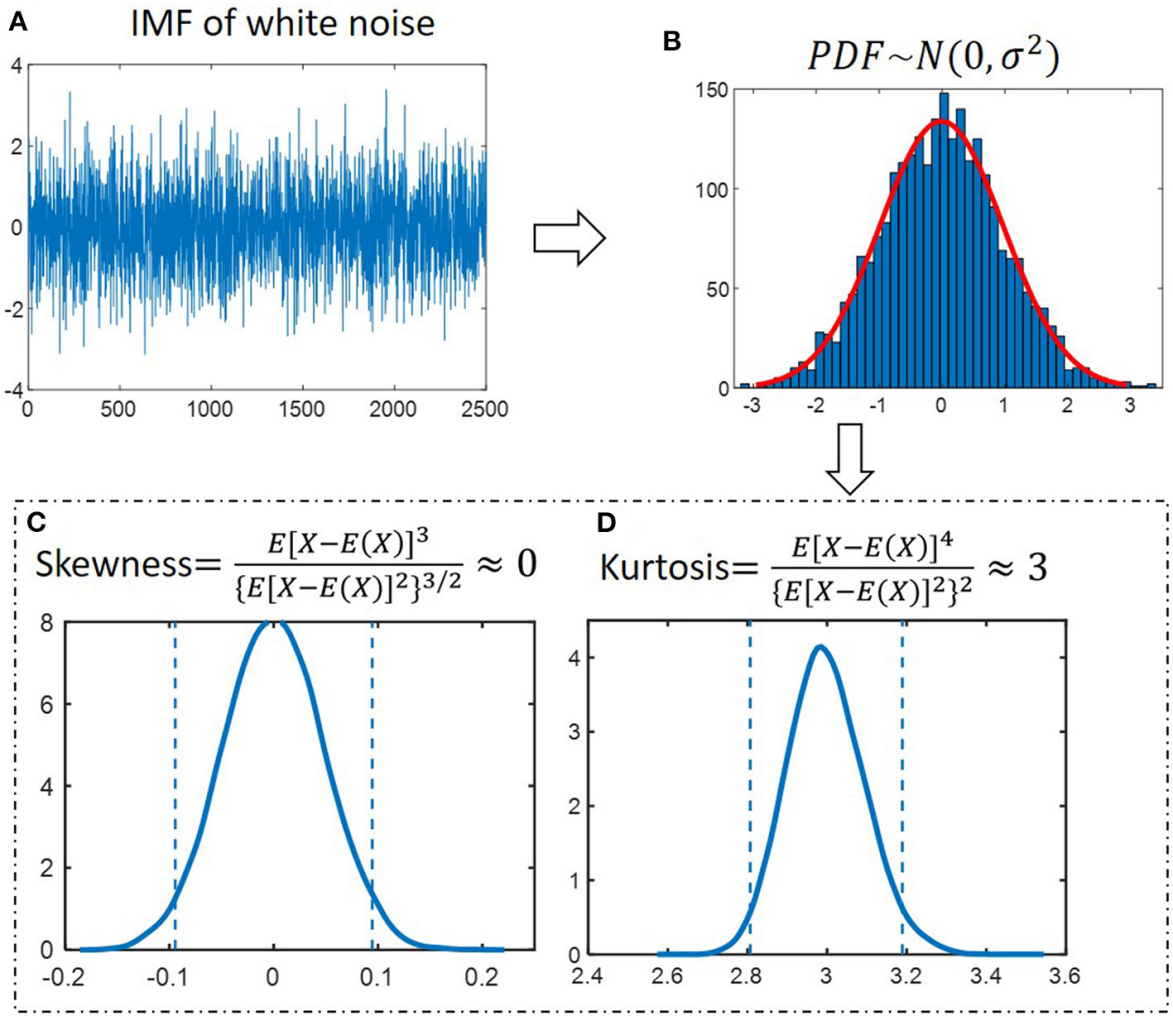
Properties of IMFs derived from white noise. **(A)** An IMF component of white noise. **(B)** The probability distribution function (PDF) of amplitude follows a normal distribution. **(C)** Skewness for 10^5^ simulated noise epochs is calculated and distributions were determined. The dashed lines at−0.09 and 0.09 depict the 95% confidence interval. **(D)** Kurtosis for 10^5^ simulated noise epochs is calculated and distributions were determined. The dashed lines at 2.8 and 3.2 depict the 95% confidence interval.

Kurtosis and skewness have been used as the criterion for noise detection or reduction, such as radio-frequency interference detection of microwave radiometers (34), voice activity detection (35), fault detection (36). Figure 3A presents an IMF component of white noise. The probability density function (PDF) of amplitude follows the normal distribution, as shown in Figure 3B. We generate 2500-points white noise for 10^5^ epochs. The kurtosis and skewness values were calculated for 10^5^ epochs. The distributions were shown in Figures 3C,D. The 0.05 and 0.95 quantiles are determined to define the lower and upper thresholds. The 95% confidence interval is [−0.09, 0.09] and [2.8, 3.2] for skewness and kurtosis, respectively.

(c) Energy-density spread function

As suggested by Wu and Huang (32), Two parameters, energy density and average period, were defined to characterize the targeted IMF. The energy density is calculated by the following equation:

(8) $$\begin{array}{l} E_{n}=\frac{1}{N}\sum_{j=1}^{N}{\left[C_{n}\left(j\right)\right]}^{2} \end{array}$$

Where *C*_*n*_ (*j*) is the *n*th IMF, *N* is the length of time-series.

The average period is derived based on the fact that all the Fourier spectra except the first one have almost identical shapes in terms of the semi-logarithmic period scale (lnT). The area coverage for each spectrum is identical. The averaged period calculated from any given spectrum is defined as:

(9) $$\begin{array}{l} \bar{T_{n}}=\int S_{\ln\;T,n}dlnT{\;\left(\int S_{\ln\;T,n}\frac{dlnT}{T}\;\right)}^{-1} \end{array}$$

where *S*_ln *T,n*_ is the Fourier spectrum of the *n*th IMF as a function of ln*T*; *T* is period. This value is almost identical to *N*/*N*_max_. *N*_max_ is the number of local maxima.

For IMFs of white-noise series, the relation between energy density and the average period is

(10) $$\begin{array}{l} \ln\;E_{n}+\ln\bar{T_{n}}=const \end{array}$$

**Figure 4:**
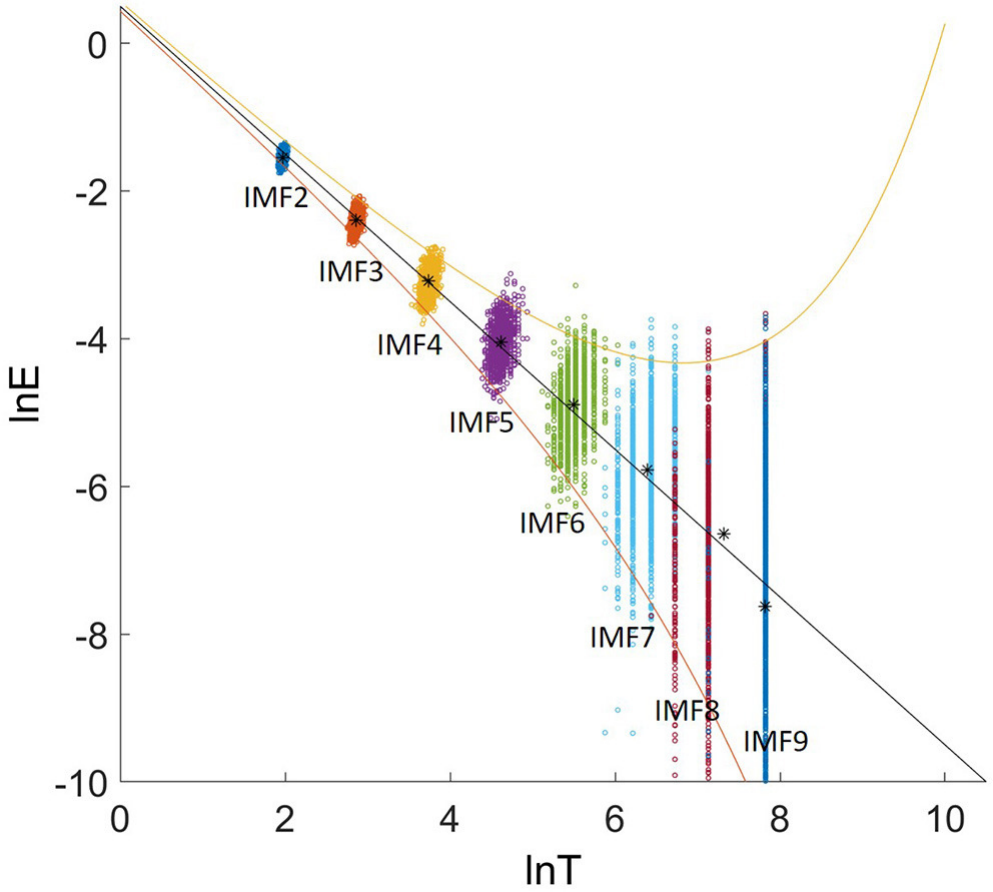
The relation between ln*E* and ln*T*. The asterisks are the mean energy density as a function of the averaged period for IMF 2-9. The upper bound and lower bound are spread line with the first and 99^th^ percentiles.

Figure 4 shows the relation between the energy density and the averaged period. The groups of dots from upper left to the lower right are the energy density as a function of the average periods for IMF 2-9 for all 1000 samples with an identical length of 2500 data points. The asterisk are the mean energy density as a function of the averaged period for IMF 2-9.

The spread line of energy and period in terms of logarithmic scale can be defined as:

(11) $$\begin{array}{l} \{\begin{array}{c} y=-x+b\pm k\sqrt{2/N}e^{x/2}\\ y=lnE_{n}\\ x=\ln\bar{T_{n}} \end{array} \end{array}$$

*k* is a constant determined by the percentiles of a standard normal distribution. *k* equal to −2.326, −0.675, 0 and 0.675, 2.326 for the first, 25^th^, 50^th^, 75^th^, and 99^th^ percentiles, respectively, *N* is the number of data points, *b* is Y-intercept. More details can be found in Wu and Huang (32).

(2) Procedure of the DEEMD

The specific steps of the DEEMD algorithm are as follows:

Step 1: Construct reference white-noise sections with identical length of EEG signal. Each white-noise section is decomposed into IMFs using EMD.
Step 2: Calculate the energy-density spread function of various percentiles for white noise. A confidence-limit level (e.g., 99%) is selected to determine the upper and lower spread lines.
Step 3: EEG signal is decomposed into IMFs using EEMD. The kurtosis and skewness of the first IMF are calculated. Compare the skewness and kurtosis for IMF1 from EEG data with the interval [−0.09, 0.09] and [2.8, 3.2]. If these two statistics fit the intervals, IMF1 is considered to be noise.
Step 4: Calculate the energy density and average period of IMF2-9. Compare the energy density with the spread functions. If the energy is located above the upper bound or below the lower bound, this IMF should be considered to contain information.

### Feature Extraction of IMFs

Researchers have shown that the statistical features of IMFs are useful in some recognition tasks. The features obtained from each IMF can give a rich clue about the physiology of the EEG signal. In this work, we extract time domain and frequency domain features from IMFs. Table 1 lists the features extracted from each IMF. The following are details of key features used in our method.

**Table 1:** List of extracted features for each IMF.

**Category**	**Feature name**	**Dimensions**
Time-domain	Mean, Standard deviation, Skewness, Kurtosis, Max, Min, First Difference, Second difference, Normalized first difference, Normalized second difference, Hjorth (Activity, Mobility, Complexity), Fractal Dimension	14
Frequency-domain	Spectral centroid, Spectrum variance Spectral skewness, Spectral kurtosis, Coefficient of variation of envelop (CVE), Raw moment of first derivative of instantaneous frequency (RMFDIF), Spectral moment of power spectral density (SMPSD).	7

#### First Difference of IMF Time Series

The first difference of times series *Dt* depicts the intensity of signal change in the time domain. Previous research has revealed that the variation of EEG time series can reflect different emotion states [2]. For an IMF component with *N* points, IMF{imf1, imf2,..., imf*N*}, the definition of *Dt* is.

(12) $$\begin{array}{l} D_{t}=\frac{1}{N-1}\sum_{n}^{N-1}|imf\left(n+1\right)-imf\left(n\right)| \end{array}$$

#### Coefficient of Variation of the Envelope

It is widely accepted that neural synchrony is associated with observable EEG fluctuations in both amplitude and morphology. Díaz et al. (37, 38) found the coefficient of variation of the envelope (CVE) is highly correlated with relevant aspects of signal morphology and can be used as a practical feature extraction method for neural signals and other bio-signals. Each IMF decomposed from the original EEG signal is both amplitude and frequency modulated. We use CVE to study the amplitude characteristics of IMFs. The Hilbert Transform is applied to obtain the envelope of each IMF. For any signal x(t), its Hilbert transform y(t) is defined as:

(13) $$\begin{array}{l} y\left(t\right)=\frac{1}{\pi }\int \frac{x\left(\tau \right)}{t-\tau }d\tau \end{array}$$

The corresponding analytical signal is:

(14) $$\begin{array}{l} q\left(t\right)=x\left(t\right)+iy\left(t\right) \end{array}$$

The envelope of x(t) was obtained using

(15) $$\begin{array}{l} env\left(t\right)=\sqrt{x^{2}\left(t\right)+y^{2}\left(t\right)} \end{array}$$

The mean and standard deviation of env were calculated to obtain CVE:

(16) $$\begin{array}{l} CVE=st\left(env\right)/mean\left(env\right) \end{array}$$

#### Raw Moment of First Derivative of Instantaneous Frequency

This feature represents the weighted successive difference of instantaneous frequency (IF) of an IMF. It asses the frequency variability characteristics of EEG signals by including extreme values. The IF from the phase of *m*th IMF and its difference are defined as:

(17) $$\begin{array}{l} f_{i}=\frac{1}{2\pi }diff\;\{\Phi_{m}\},\;\delta_{f}=|diff\;(f_{i})| \end{array}$$

The RMFDIF feature is computed as:

(18) $$\begin{array}{l} \text{RMFDIF}=\frac{1}{N-1}\sum_{n=1}^{N-1}\delta_{f}\left[n\right] \end{array}$$

Where N is the number of the samples in IF.

#### Spectral Moment of Power Spectral Density

Welch's method is used in the computation of PSD. The PSD of an analytic IMF *q*(*t*) is represented as:

(19) $$\begin{array}{l} S_{q}\left(f\right)=\underset{T\to \infty }{\text{lim}}\left\{\frac{1}{2T}|\sum_{n=-T}^{T}q\left(t\right)e^{-j2\pi fn}{|}^{2}\right\} \end{array}$$

The spectral moment of PSD is used to define the greater order shape of EEG signal, which can be defined as:

(20) $$\begin{array}{l} SMPSD=\sum_{k=1}^{L}k.PSD_{k} \end{array}$$

Where L is the number of points in PSD.

### Spatial Pyramid Pooling Network

With throwing noise-dominant IMF components out, the number of remaining IMFs varies between different samples. Therefore, the size of feature maps is arbitrary. However, most of the classifiers (SVM/softmax) or fully-connected layers require fixed-size/length input by their definition.

In this article, we employ the SPP-net (39) as the classifier to recognize emotions. SPP-net is inspired by the Bag of Words approach (40). It is one of the most successful methods in computer vision and object detection. SPP-net adds a pyramid pooling layer after the last convolution layer. The pyramid pooling layer can transform any size feature map into a fixed-size feature vector. This layer also aggregates local features from finer to coarser levels. By multi-level spatial pooling, it can enhance the robustness of the network and improve detection accuracy. SPP-net has several remarkable advantages for addressing the issue mentioned in the previous section: (1) SPP-net can generate a fixed-length representation from arbitrarily sized input, then match with full connection layer. In SPP-net, the number of bins for pooling is fixed instead of the fixed sliding window size. (2) Multi-level spatial pooling can not only maintain spatial information but also is robust to the variance in spatial layout (39, 41). In the following, we describe the proposed network in detail.

#### Feature Processing

**Figure 5:**
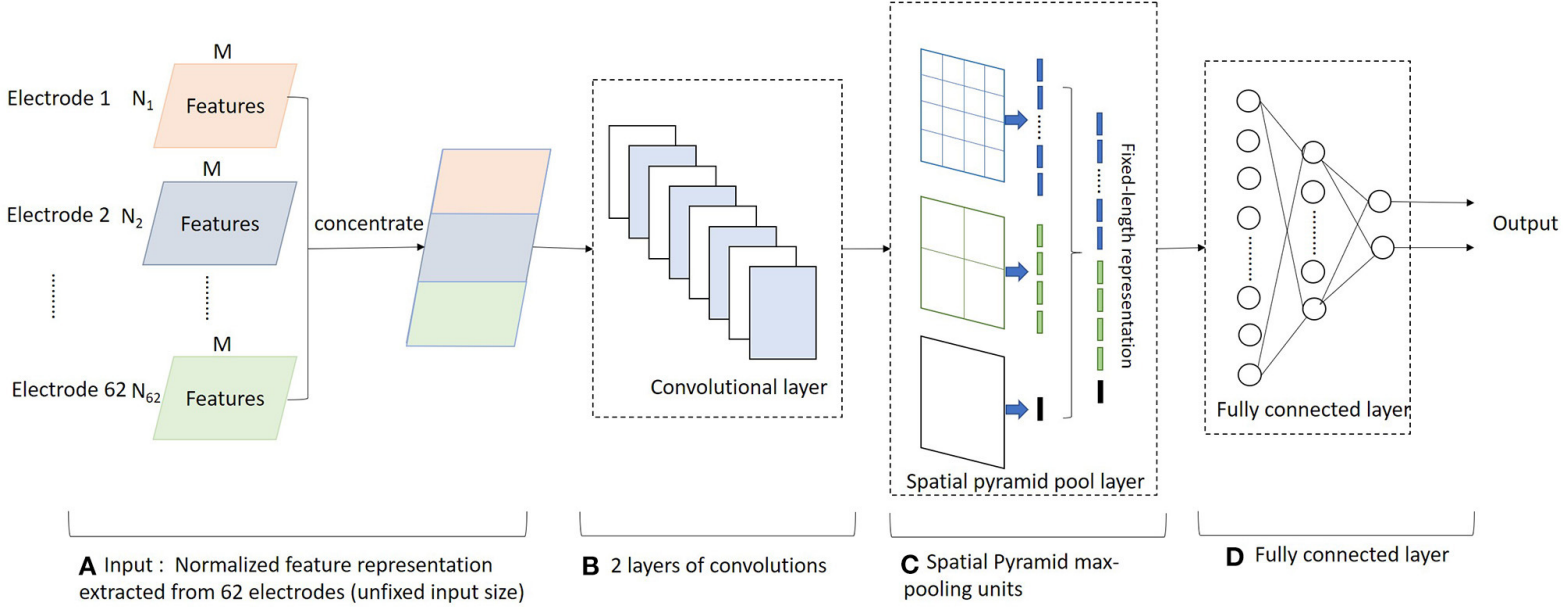
The architecture of the SPP-net model. **(A)** Feature processing. **(B)** Convolutional layers. **(C)** Spatial pyramid pool layer. **(D)** Fully connected layer.

For the *i-*th electrode, the EEG signal is decomposed by DEEMD. We extract time domain and frequency domain features from the selected IMFs. The features are listed in Table 1. We obtain a feature descriptor **f**_*i*_ of dimensionality (*N*_*i*_, *M*). *N*_*i*_ is the number of IMFs from the *i-*th electrode EEG, *M* is the number of features. Features of all electrodes are concentrated and then normalized as the global feature representation (Figure 5A).

#### Network Architecture

The SPP-net consists of an array of CNN subnet, a spatial pyramid pooling layer, and fully connected layers, as shown in Figures 5B–D. Convolution operations do not require the fixed input size, but the fully connected layer requires a fixed dimension. The pyramid pooling layer can transform any size feature map into a fixed-size feature vector. The CNN subnet consists of two convolution layers with kernel sizes of 10 × 4 and 5 × 2, respectively.

In the SPP layer, we use parallel max-pooling layers at several levels. We implement these pooling levels by sliding windows of different sizes. Considering an *l*-level pyramid of *n*_*l*_ × *n*_*l*_ bins, the sliding window size is *win* = ⌈*a*/*n*_*l*_⌉ and stride is *str* = ⌊*a*/*n*_*l*_⌋. The symbols ⌈.⌉ and ⌊.⌋ denote ceiling and floor operations. The responses of all levels are then concatenated to get a fixed-length feature vector of size $\sum_{l}n_{l}\times n_{l}$. The combination of different levels can not only detect large-scale feature change areas, but also the small details, which makes the network more flexible and robust.

The fixed-size feature vector is then fed into three subsequent fully connected layers.

## Results and Discussion

### Dataset

Speech carries emotional information in human communication. In this article we consider a dataset collected from a speech-evoked emotion cognitive experiment, with full description in Chen et al. (10). Nineteen healthy participants (8 females and 11 males) with a mean age of 22.4 years (ranging between 18 and 27 years) participated in the experiment. The stimuli were 5-s audio clips without background sound. Each clip contains at least a complete utterance. The discrete affective label and dimensional emotional annotation (Arousal-Valence-Dominance) with 1-9 scales related to each stimulus were obtained using Amazon's Mechanical Turk. Stimuli were presented in random order. Each trial consisted of three steps: (1) A 3 s baseline recorded; (2) A 5 s audio clip played; (2) A 30 s Self-assessment for arousal, valence, and dominance. There are two sessions during the experiment, each session consisted of 40 trials. This resulted in 80 trials total per participant. For all participants, there are a total of 1,373 trials that exclude “bad” trials.

The EEG signals were continuously sampled at 1,000 Hz using a 62-channel EEG system. The electrodes were placed over the scalp according to the international 10–20 system. The signal pre-processing was performed. The EEG signals were average referenced, down-sampled to 500 Hz, and filtered with 1–49 Hz to obtain the desired frequency range and remove the electrical line noise. Independent component analysis (ICA) was used to remove eye artifacts. And 3 s baseline before the audio clip was removed to correct stimulus-unrelated variations.

### The Influence of Added Noise in EEMD

From the EEMD procedure, it is obvious that the number of the ensemble and the noise amplitude are the two prescribed parameters. The residue of added white noises should be reduced following the statistical rule:

(21) $$\begin{array}{l} \varepsilon_{n}=\frac{\varepsilon }{\sqrt{N}} \end{array}$$

**Figure 6:**
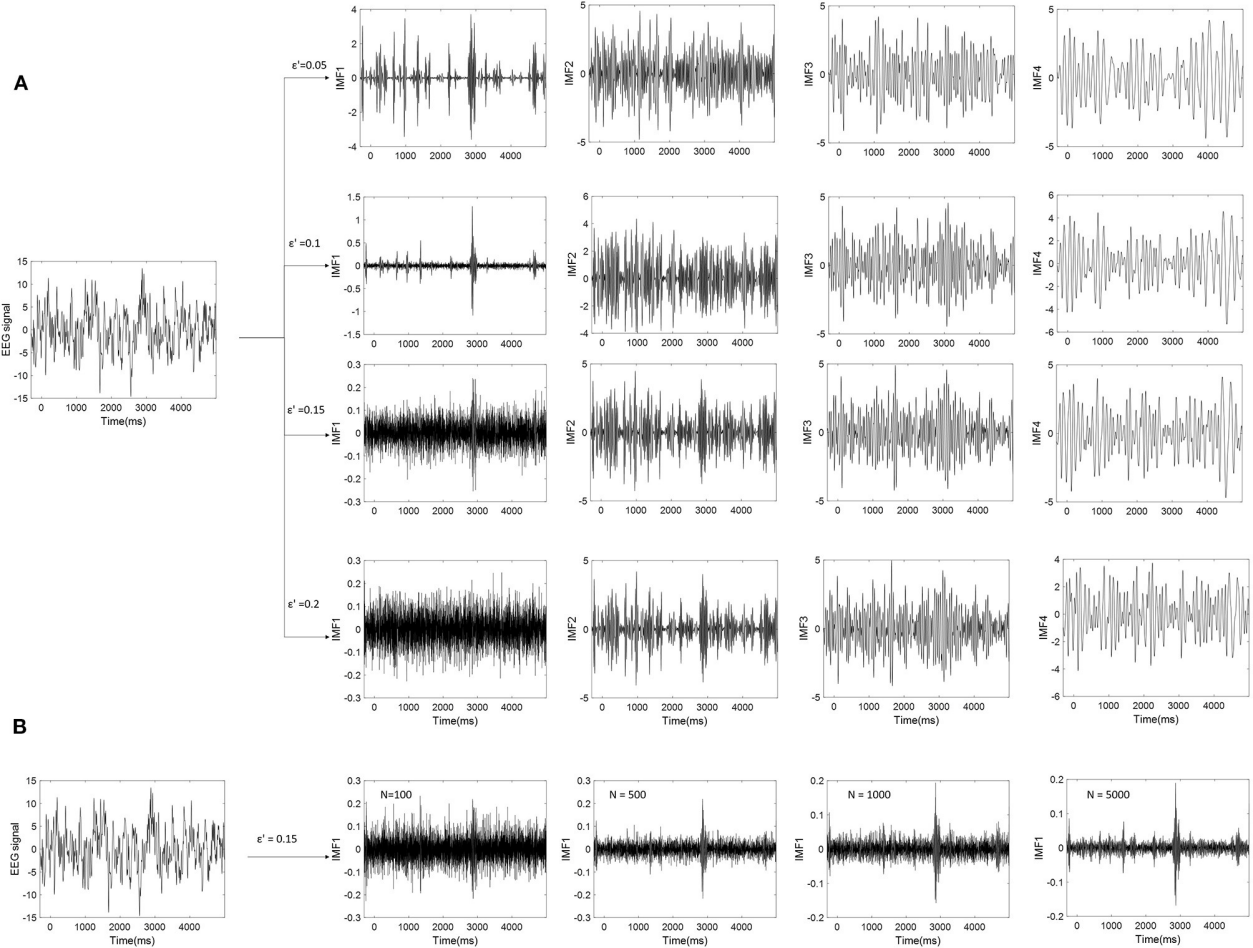
**(A)** The EEMD decomposition of EEG signal by adding different Gaussian noise. **(B)** The effect of noise on the first IMF (IMF1) are reduced by increasing the number (N) of ensemble members (ε′ is set to 0.15).

Where ε_*n*_ is the final standard deviation of error; ε is the amplitude of the added noise; and *N* is the number of ensemble members. To make the EEMD effective, the amplitude of the added noise could not be too small. Because it may not introduce the change of extrema when the noise amplitude is too small, especially for the data with a large gradient. However, if the amplitude of the noise is large enough, the number of ensemble members should be increased to reduce the effect of noise. At the same time, it also causes higher computation costs. Figure 6 presents the relation in equation (21). It depicted the results of EEMD decomposition of an EEG signal during one trial. ε′ is the ratio of the standard deviation of the added noise and that of the original EEG signals. From Figure 6A, it can be seen that the first IMF component is easily influenced by noise, followed by the second IMF. As the amplitude of the added noise increase, the amplitude of the first IMF decrease. The first IMF may be a noise-dominant component. Figure 6B shows that the effect of noise can be reduced to a negligibly small level by increasing the ensemble members. This example shows that not all of the obtained IMF components are valuable for EEG analysis. Some IMFs are noisy or did not carry valuable information.

### Validation of Selected IMFs Using DEEMD

**Figure 7:**
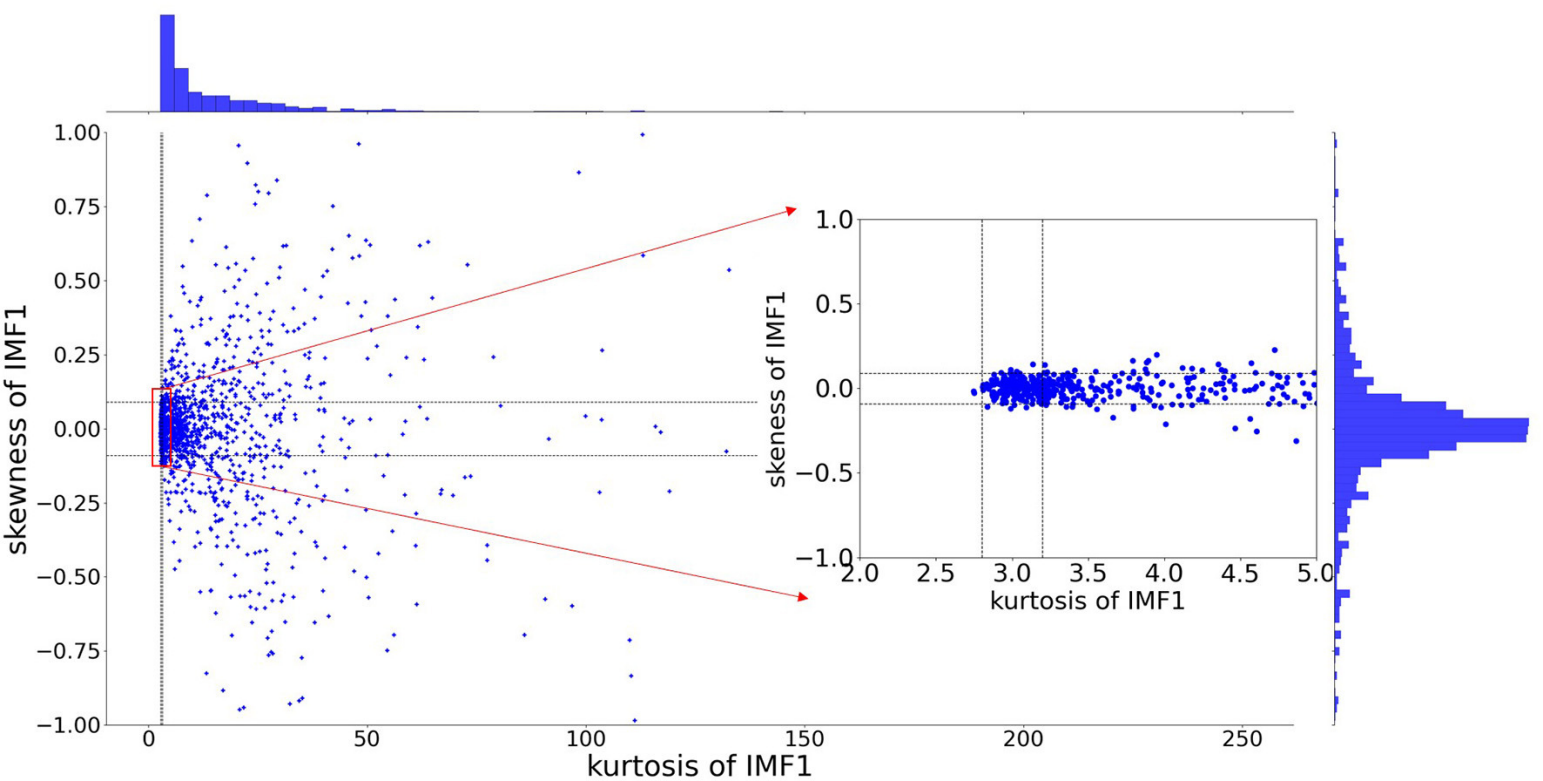
The distribution of skewness and kurtosis for the first order IMF extracted from EEG signal (at PF1 electrode for all 1,373 trials).

As discussed in Section The Influence of Added Noise in EEMD, the extracted IMFs can be either signal-dominant or noise-dominant. It is crucial to select informative IMFs that contain intrinsic information about brain activity. This article presents an adaptive selection criterion for informative signal-dominant IMF. To define the evaluation criterion, we have analyzed the amplitude and frequency properties of IMFs for white noise. In this study, EEG signals in a speech evoked emotion cognitive experiment are studied. The preprocessed EEG signals are decomposed through EEMD. The number of ensemble members is set as 1,000. The ratio of the standard deviation of the added noise to that of the raw signal is 0.3. Then we calculated the skewness and kurtosis of the IMF1. Figure 7 demonstrates the distributions of skewness and kurtosis for the first order IMF component. These IMF1 are derived from the FP1 electrode for all 1,373 trials. 12.7% IMF1 has skewness between −0.09 and 0.09, and kurtosis between 2.8 and 3.2.

**Figure 8:**
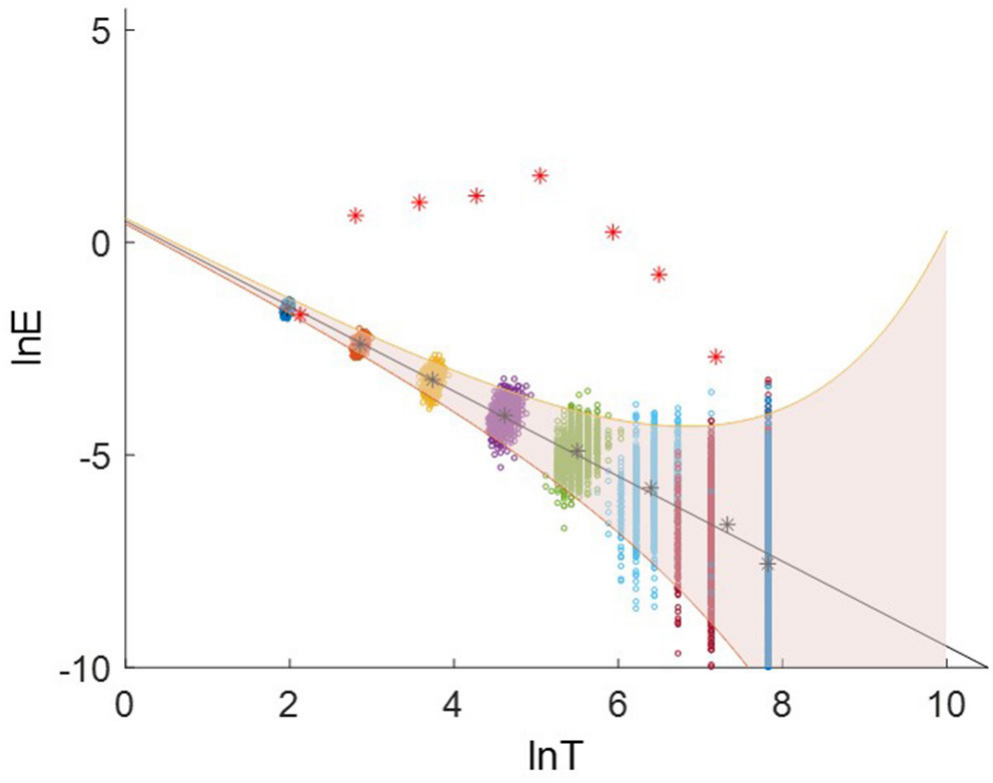
Logarithmic energy density-averaged period plot. The red asterisks are the distribution for the IMF2-9 decomposed from an EEG signal. The groups of dots present the distribution for the white-noise series.

We generate 1000 white-noise series as the reference samples. Each sample contains 2500 data points with the identical length as the targeted EEG signal. These samples are decomposed using EMD. The averaged period and energy density of IMF 2-9 are plotted in Figure 8. The groups of dots from the upper left to the lower right are the energy density as a function of the average periods for IMF 2-9. The black solid line is the theoretical expectation of the pair of averaged period and energy. The upper (the 99^th^ percentiles) and lower bound (the first percentiles) are determined from the probability distribution of the energy density of the IMFs from Gaussian white noise.

For each EEG signal, we calculated the energy densities and average periods of IMF 2-9. They are compared with the reference white-noise samples to determine whether a specific IMF contains significant information. The red asterisks in Figure 8 are the energy density vs. corresponding average periods for the IMF2-9 from an EEG signal. The IMF2 presents a distribution similar to the result from white noise. Therefore, IMF2 was identified as the noisy component. IMF 3-9 shows a higher energy level than that of white noise. They are above the significant limit for white noise, therefore, they are identified as the signal-dominant components. The Supplementary Figure 1 plots the distribution of energy density vs. corresponding average periods of EEG signals for 80 trials.

**Figure 9:**
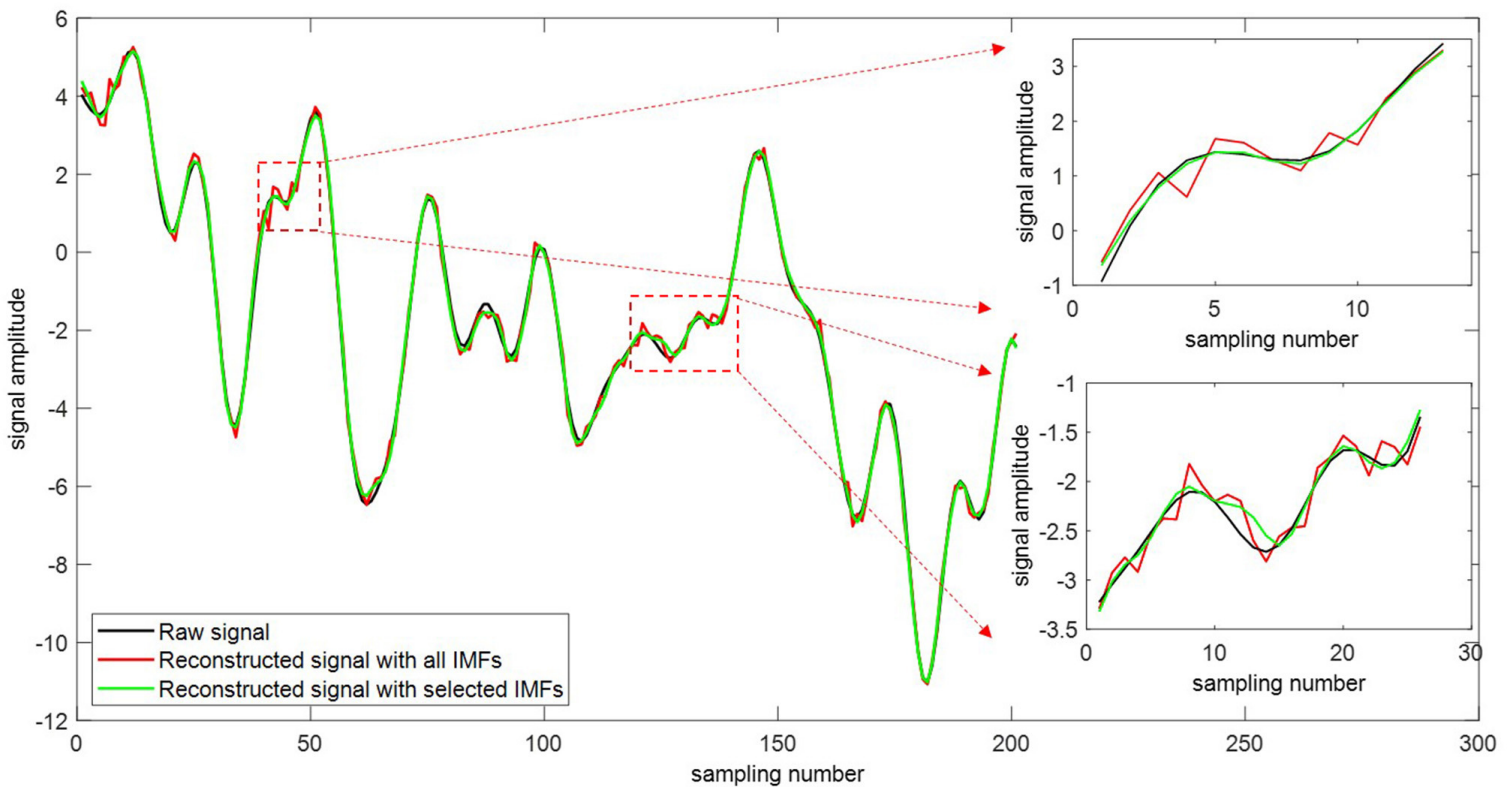
Comparison of time-domain signals with reconstructed signals.

The original signal can be reconstructed using the extracted intrinsic modes and the residue signal (Equation 5). Figure 9 depicts the comparison of the original signals with the reconstructed ones. The red curve is the reconstructed signal with all IMFs. The green one is the reconstructed signal with selected sensitive IMFs. As the added noise cannot be filtered completely, spurious modes are generated due to the residual noise. The impact of spurious mode mainly appears in the local extremum. It is seen that our proposed selection method delivers a more reconstructed signal and improves the reconstruction accuracy.

Furthermore, to quantitively evaluate the quality of signals reconstructed after the IMF selection, the signal-to-noise ratio (SNR) and mean square error (MSE) metrics are employed in this study.

(22) $$\begin{array}{l} MSE=\;\frac{1}{N}\sum_{n=1}^{N}{\left(x\left(n\right)-x_{rec}\left(n\right)\right)}^{2} \end{array}$$

(23) $$\begin{array}{l} SNR=\;10\log_{10}\left(\frac{\sum_{n=1}^{N}{\left(x\left(n\right)\right)}^{2}}{\sum_{n=1}^{N}{\left(x\left(n\right)-x_{rec}\left(n\right)\right)}^{2}}\right) \end{array}$$

**Figure 10:**
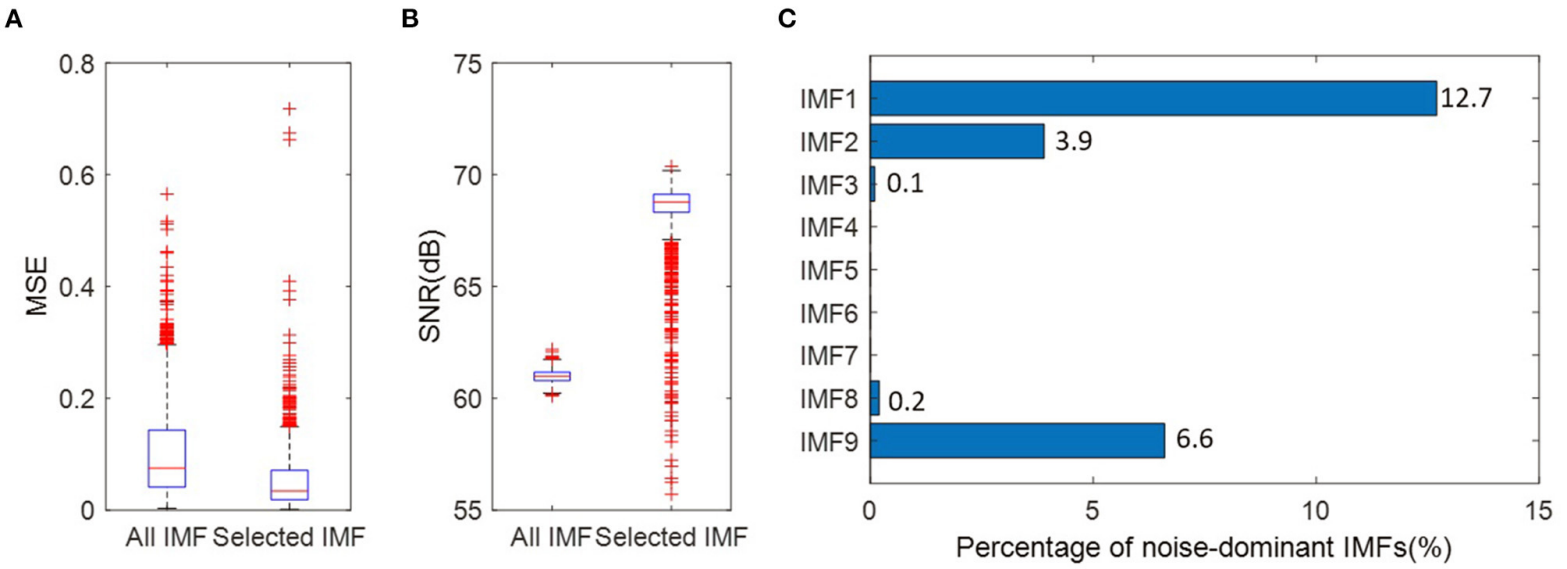
**(A)** MSE between the reconstructed signals and the original signal. The reconstructed signals are obtained by all IMFs or selected IMFs, respectively. **(B)** SNR between the reconstructed signals and the original signal. **(C)** The percentage of noise-dominant IMFs at the “PF1” electrode for 1,373 trials.

Where *x* (*n*) is the original signal and *x*_*rec*_(*n*) is the reconstructed signal. N is the length of data points. The smaller MSE and bigger SNR exhibit higher reconstruction accuracy and better quality of the reconstructed signal. Figures 10A,B present the comparison between the reconstructed signals with the original signals. The original signals are EEG signals at “FP1” electrode from 1373 trials. The reconstructed signals are obtained by all IMFs or selected IMFs, respectively. Figure 10A shows that constructed signals using the selected IMFs have relatively smaller MSE. It demonstrates that IMF selection allows the reduction of the noise for reconstructed signals. From Figure 10B, one can observe that constructed signals using the selected IMFs demonstrate significantly better results in terms of the SNR. Figure 10C presents the percentage of noise-dominant IMFs.

### Emotion Recognition

The performance of the proposed framework DEEMD-SPP for EEG-based emotion recognition is studied in this subsection. We evaluate the prediction accuracy in the level of valence and arousal separately. Among the total 1,373 trials of all subjects, 790 trials are labeled as high valence and 583 as low valence. For arousal, there are 815 trials as high arousal and 558 trials as low arousal.

For each trial, the EEG signal of each electrode is firstly decomposed by DEEMD. The IMF selection process of DEEMD provides meaningful IMFs that carry important information in the original signal. Then we extract time domain and frequency domain features from each selected IMF. The dimension of features is 21. The features of 62 electrodes are concentrated to a feature representation. The size of the feature representation matrix is approximately between 610 × 21 and 623 × 21. In the last step, we apply SPP-net to process arbitrarily sized input and aggregate information at a multi-level. The parameters used in our model are demonstrated in Table 2.

**Table 2:** Architecture and parameter settings of SPP-net.

**Layer type**	**Input size**	**Output size**	**Patch size**	**Kernel**	**Stride**
1^st^ Convolution layer	(610~623) × 21	(121~128) × 18 × 10	(10.4)	10	(5.1)
2^nd^ Convolution layer	(121~128) × 18 × 10	(117~124) × 18 × 200	(5.2)	20	(1.1)
SPP layer	(117~124) × 18 × 200	4,200	[4, 2, 1]	-	-
1^st^ Fully-connected layer	4,200	500	500	-	-
2^nd^ Fully-connected layer	500	200	200	-	-
Output layer	200	2	2	-	-

A 5-fold cross-validation method has been adopted for performance evaluation. We split the entire dataset, which has 1373 trials, into 5 folds. In each iteration, 1-fold (275 trials) is used to test the model and the rests (1,098 trials) serve as the training set. The process is repeated until each fold of the 5-folds has been used as the training set.

For a two-class classification problem, the accuracies are measured using

(24) $$\begin{array}{l} Accuracy=\;\frac{TP+TN}{TP+TN+FN+FP} \end{array}$$

where TP, TN, FP, FN denote true positive, true negative, false positive, false negative, respectively.

To assess the proposed method, five experiments are conducted on the dataset. In the first experiment, we decompose the EEG signals using EEMD without an IMF selection procedure. Features are extracted from each IMFs (Table 1). Then the statistics (mean, standard deviation, 25 and 75% quantiles) of these features for all IMFs are calculated as input to SVM. In the second experiment, we decompose the EEG signals using EEMD. Features are extracted from the first five IMFs. The statistics of these features are input to SVM. In the third experiment, we decompose the EEG signals using EEMD without an IMF selection procedure. Features are extracted from all IMFs. These features are input to SPP-net. In the fourth experiment, we decompose the EEG signals using DEEMD. The IMFs are selected self-adaptively. Features are extracted from the selected IMFs. The statistics of these features are calculated as input to SVM and ANN, respectively. In the fifth experiment, our proposed DEEMD-SPP framework is used. The results of these experiments are given in Table 3. Comparing the results obtained from the first three experiments show that features extracted from each IMF perform better than statistics. Statistics will lose important information due to a high degree of abstraction. The statistics from the first five IMFs do not necessarily have higher accuracy. This is possibly because the relationship between each IMF with EEG rhythm can differ depending on the frequency and the possible noise effects. Further experiments show that our proposed DEEMD-SPP framework has the best performance with 74.5 and 72.2% accuracy for valence and arousal, respectively. The accuracy by IMF selection using DEEMD exceeds the one of the third experiment by ~2% for valence and arousal.

**Table 3:** The classification accuracies of valence and arousal.

**Method**	**IMF**	**Features**	**Classifier**	**Accuracy**	
				**Valence (%)**	**Arousal (%)**
EEMD	All IMFs	Statistics	SVM	70.8	68.4
	First five IMFs	Statistics	SVM	68.6	69.3
	All IMFs	Features in Table 1	SPP-net	72.1	70.6
DEEMD	Selected IMFs	Statistics	SVM	69.7	68.1
	(proposed)	Statistics	ANN	70.2	68.8
		Features in Table 1	SPP-net (proposed)	**74.5**	**72.2**

*The bold value means the best performance*.

## Conclusion

EEG-based emotion recognition is a growing research field of affective computing. It requires accurate and efficient signal processing and feature extraction methods. In this article, we propose a novel framework named DEEMD-SPP to improve the accuracy and effectiveness of emotion recognition based on EEG. DEEMD-SPP uses a novel feature extraction method named denoising ensemble empirical mode decomposition (DEEMD) and Spatial Pyramid Pooling Network (SPP-Net) for classification. The framework contains three steps. First, DEEMD is proposed to decompose the EEG signals and select the most valuable IMFs. Second, time domain and frequency domain features are extracted from the selected IMFs. Finally, SPP-Net is employed as the classifier to recognize emotions. To demonstrate the advantages of DEEMD-SPP, we first investigate the IMF selection capabilities of the proposed DEEMD, using an EEG dataset collected from a speech-evoked emotion cognitive experiment. The experimental results demonstrate that the IMF selection procedure of DEEMD allows for the better exclusion of the noise-dominant components. Additionally, we compare our proposed framework with four state-of-the-art methods on EEG-based emotion recognition. The experiments show that our method achieves higher accuracy than the other methods, indicating that the proposed learning-based framework is appropriately designed. The proposed DEEMD-SPP framework will benefit the studies in psychology, psychiatry, and public health that involve EEG-based affective analysis.

## Data Availability Statement

The original contributions presented in the study are included in the article/Supplementary Material, further inquiries can be directed to the corresponding author/s.

## Author Contributions

JC was involved in experiment conduction, data analysis, and manuscript write up. HL, LM, and FS were involved in the conception, supervision, and manuscript review. All authors contributed to the article and approved the submitted version.

## Funding

This research was supported by the National Natural Science Foundation of China (U20A20383), National Key Research and Development Program of China (2020YFC0833204), Shenzhen Foundational Research Funding (JCYJ20200109150814370), Basic and Applied Basic Research of Guangdong (2021A1515011903 and 2021B1515120052), and Heilongjiang Touyan Team.

## Conflict of Interest

FS was employed by company Microsoft Research Asia. The remaining authors declare that the research was conducted in the absence of any commercial or financial relationships that could be construed as a potential conflict of interest.

## Publisher's Note

All claims expressed in this article are solely those of the authors and do not necessarily represent those of their affiliated organizations, or those of the publisher, the editors and the reviewers. Any product that may be evaluated in this article, or claim that may be made by its manufacturer, is not guaranteed or endorsed by the publisher.
