# Supplementary material for: DEEMD-SPP: A Novel Framework for Emotion Recognition Based on EEG Signals
Source: Front Psychiatry. 2022 Apr 27;13:885120. doi: 10.3389/fpsyt.2022.885120 (PMC9091650; doi:10.3389/fpsyt.2022.885120)
Supplement: Supplementary file 1 [file Image_1.pdf]

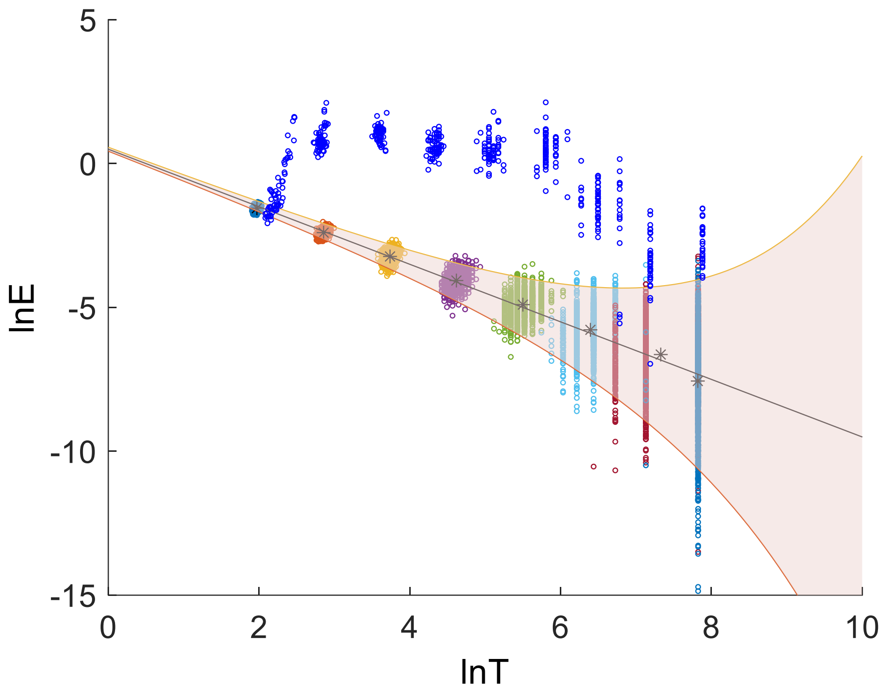

Figure S1. Logarithmic energy density-averaged period plot. The groups of blue dots are the distribution of the IMF2-9 decomposed from EEG samples (at PF1 electrode for 80 trials).
